# Mice with an *N*-Ethyl-*N*-Nitrosourea (ENU) Induced Tyr209Asn Mutation in Natriuretic Peptide Receptor 3 (NPR3) Provide a Model for Kyphosis Associated with Activation of the MAPK Signaling Pathway

**DOI:** 10.1371/journal.pone.0167916

**Published:** 2016-12-13

**Authors:** Christopher T. Esapa, Sian E. Piret, M. Andrew Nesbit, Nellie Y. Loh, Gethin Thomas, Peter I. Croucher, Matthew A. Brown, Steve D. M. Brown, Roger D. Cox, Rajesh V. Thakker

**Affiliations:** 1 Oxford Centre for Diabetes, Endocrinology and Metabolism, Radcliffe Department of Medicine, University of Oxford, Oxford, United Kingdom; 2 MRC Mammalian Genetics Unit and Mary Lyon Centre, MRC Harwell, Harwell Science and Innovation Campus, Harwell, United Kingdom; 3 Institute of Health and Biomedical Innovation, Queensland University of Technology, Translational Research Institute, Princess Alexandra Hospital, Brisbane, Queensland, Australia; 4 Garvan Institute for Medical Research, Sydney, Australia; Charles P. Darby Children's Research Institute, 173 Ashley Avenue, Charleston, SC 29425, USA, UNITED STATES

## Abstract

Non-syndromic kyphosis is a common disorder that is associated with significant morbidity and has a strong genetic involvement; however, the causative genes remain to be identified, as such studies are hampered by genetic heterogeneity, small families and various modes of inheritance. To overcome these limitations, we investigated 12 week old progeny of mice treated with the chemical mutagen *N*-ethyl-*N*-nitrosourea (ENU) using phenotypic assessments including dysmorphology, radiography, and dual-energy X-ray absorptiometry. This identified a mouse with autosomal recessive kyphosis (KYLB). KYLB mice, when compared to unaffected littermates, had: thoraco-lumbar kyphosis, larger vertebrae, and increased body length and increased bone area. In addition, female KYLB mice had increases in bone mineral content and plasma alkaline phosphatase activity. Recombination mapping localized the *Kylb* locus to a 5.5Mb region on chromosome 15A1, which contained 51 genes, including the natriuretic peptide receptor 3 (*Npr3*) gene. DNA sequence analysis of *Npr3* identified a missense mutation, Tyr209Asn, which introduced an N-linked glycosylation consensus sequence. Expression of wild-type NPR3 and the KYLB-associated Tyr209Asn NPR3 mutant in COS-7 cells demonstrated the mutant to be associated with abnormal N-linked glycosylation and retention in the endoplasmic reticulum that resulted in its absence from the plasma membrane. NPR3 is a decoy receptor for C-type natriuretic peptide (CNP), which also binds to NPR2 and stimulates mitogen-activated protein kinase (MAPK) signaling, thereby increasing the number and size of hypertrophic chondrocytes. Histomorphometric analysis of KYLB vertebrae and tibiae showed delayed endochondral ossification and expansion of the hypertrophic zones of the growth plates, and immunohistochemistry revealed increased p38 MAPK phosphorylation throughout the growth plates of KYLB vertebrae. Thus, we established a model of kyphosis due to a novel NPR3 mutation, in which loss of plasma membrane NPR3 expression results in increased MAPK pathway activation, causing elongation of the vertebrae and resulting in kyphosis.

## Introduction

Kyphosis, a common disorder in humans, is characterized by excessive curvature of the vertebral column [1] that can occur at any age. Kyphosis may result from trauma, metabolic disorders, neuromuscular diseases, vertebral fusion [2, 3], and osteoporotic fractures [4]. In addition, genetic diseases may give rise to kyphosis in isolation or in association with other developmental abnormalities, such as Larsen Syndrome (OMIM: #150250), due to dominant mutations in *FLNB* [5, 6]. The most common form of adolescent isolated kyphosis, referred to as Scheuermann disease (OMIM: %181440), affects >8% of the population and may be inherited in an autosomal dominant manner, although the causative gene(s) remains to be identified [7–10]. A study of adult female twins reported the heritability of thoracic kyphosis to be >60%, thereby demonstrating that kyphosis has a strong genetic component [11].

Identification of the genetic abnormalities associated with isolated forms of kyphosis has been hampered by genetic heterogeneity, small families that do not enable localization of the disease locus by linkage studies, variable modes of inheritance, and gene-environment interactions that may modify vertebral phenotypes [6]. In addition, studies of the underlying mechanisms of kyphosis have been hampered by a lack of suitable models with relevance to kyphosis in humans.

To overcome these limitations and facilitate further mechanistic studies, we sought to establish mouse models for kyphosis using phenotypic assessments including dysmorphology, radiography and dual energy X-ray absorptiometry (DXA), of progeny of mice treated with the chemical mutagen *N*-ethyl-*N*-nitrosourea (ENU). ENU is a powerful alkylating agent that introduces mainly point mutations into the genome, and has been successfully used to identify mouse models for many different disorders, including skeletal dysplasias [12, 13], deafness [14], and type 2 diabetes [15, 16]. For example, an ENU-induced mutation in the collagen 2 alpha 1 (*Col2a1*) gene has been described to result in spondyloepiphyseal dysplasia congenita (SEDC), with affected mice displaying short humeri, abnormal vertebrae and disorganized growth plates [17]. Here, we report studies of an ENU-induced mouse model for autosomal recessive kyphosis, designated KYLB, that harbors a novel mutation in natriuretic peptide receptor 3 (*Npr3*).

## Materials and Methods

### Ethics statement

All animal studies were carried out using guidelines issued by the Medical Research Council in 'Responsibility in the Use of Animals for Medical Research' (July 1993) and under Home Office Project License Number 30/2433. Experiments were approved by the Medical Research Council Harwell and UK Home Office ethics committees.

### Generation of mutant mice

Male BALB/c mice were treated with ENU and mated with untreated BALB/c female mice [18]. The male progeny (G1) were subsequently mated with normal BALB/c females to generate G2 progeny. The female G2 progeny were then backcrossed to their G1 fathers, thereby facilitating autosomal recessive traits to manifest in the resulting G3 progeny [18]. These G3 progeny were assessed for skeletal phenotypes using dysmorphology for gross anatomical defects and radiography for bone abnormalities. Mice with phenotypic abnormalities were bred onto the C57BL/6J background for inheritance testing and mapping studies. Mice were fed an expanded rat and mouse no. 3 breeding diet (Special Diets Services, Witham, UK) containing 1.15% calcium, 0.82% phosphate and 4088.65 units/kg vitamin D, and given water ad libitum.

### Dysmorphology, radiography and dual-energy X-ray absorptiometry (DXA)

A simple dysmorphology screen based on observation for any gross anatomical changes was used to monitor all mice at 3–6 weeks of age, as previously described [12]. Anaesthetised mice were subjected to digital radiography at 26kV for 3 seconds using a Faxitron MX-20 digital X-ray system (Faxitron X-ray Corporation, Lincolnshire, USA) [17] and DXA using a Lunar PIXImus densitometer (GE Healthcare, Chalfont St Giles, UK) [19]. X-ray images were processed using the DicomWorks software (http://www.dicomworks.com/) [17] and DXA images were processed using the PIXImus software [19].

### Skeletal preparation and staining with Alcian blue and Alizarin red

After removal of skin, skeletons were fixed in 95% ethanol, stained with Alcian blue (8GX) for 24 hours, differentiated in 95% alcohol, treated with 1% potassium hydroxide for 2 days, washed overnight in running tap water, stained in aqueous Alizarin red S for 2 hours, washed in running tap water for 30 minutes, decolourised in 20% glycerine/1% potassium hydroxide for 1 week, and dehydrated through graded alcohols, as described [17]. Images were acquired using a Nikon D3 camera [17]. Femoral lengths were measured using electronic calipers after dissection.

### Mapping, DNA sequencing and genotyping

Genomic DNA was extracted from ear or tail biopsies as described [17]. For genome-wide mapping, DNA was amplified by PCR using a panel of 91 single nucleotide polymorphic (SNP) loci arranged in chromosome sets, and the products were analysed by pyrosequencing [20]. DNA sequencing analysis was undertaken using gene-specific primers that amplified the exons and adjacent splice sites and BigDye terminator reagents with products analysed on an ABI3100 sequencer (Life Technologies, Carlsbad, CA, USA) [17]. Genotyping was performed by PCR amplification of exon 1 using Taq PCR Mastermix (Qiagen, Crawley, UK) and the PCR products digested with restriction endonuclease Tsp509I, and separated by agarose gel electrophoresis. Images were acquired using a GelDoc^™^ UV transilluminator (Bio-Rad, Hemel Hempstead, UK) [17].

### *In vitro* expression studies of wild-type and mutant NPR3

Three ENU-induced NPR3 mutants, which are associated with kyphosis, comprising the Tyr209Asn mutation identified by this study, and the His168Asn and Ile384Asn mutations identified by previous studies [21, 22] were investigated, as follows. A full length wild-type *Npr3* cDNA was obtained from an IMAGE clone (ID: 4019152, Geneservice) and sub-cloned in-frame into the EcoRI/SacII sites of the enhanced green fluorescent protein (pEGFP)-N1 plasmid (Clontech, Saint-Germain-en-Laye, France) in two steps. In the first step, an EcoRI/PstI fragment containing 1434bp of the 1611bp of *Npr3* coding cDNA (5’ end) was digested from the IMAGE clone (5’ end) and inserted into pEGFP-N1 using corresponding restriction sites. In the second step, a 648bp PCR product was amplified from the IMAGE clone using Pfu Ultra^™^ II DNA polymerase (Agilent Technologies, Stockport, UK) to include the internal PstI site, and the remaining 3’ *Npr3* cDNA, whilst also introducing a 3’ overhang containing a SacII site by using the primers Npr3-992F (ACTGAGGACCGTGAAACCTG), and SacII-Npr3-1640R (CCGCGGAGCCACCGAAAAATGTGATCT). This PCR product was subcloned into the PstI/SacII sites of the pEGFP-N1 containing the 5’ fragment of *Npr3*. *Npr3* mutations were introduced using site-directed mutagenesis with the following forward primers: Tyr209Asn: 5’-AACTCGAGAGGAACTGT**A**ATTTCACC-3’; His168Asn: 5’-GACACGGAATACTCG**A**ACCTCACGCGCGTG-3’; and Ile384Phe: 5’-GATGGGGGGAAAATC**T**TCCAGCAGACTTGG-3’; and their reverse complement primers. Constructs were verified by DNA sequencing. The cDNA constructs (1μg of each) were transfected into COS-7 cells using jetPEI reagent (Source Bioscience, Nottingham, UK) and Western blot and immunofluorescence analyses undertaken. Western blot analyses were performed using untreated or enzymatically deglycosylated lysates from transfected COS-7 cells [19]. Briefly, lysates were boiled for 10 min in denaturing buffer (0.5% SDS, 1% 2-mercaptoethanol), and treated with endoglycosidase (Endo) H (New England Biolabs, Ipswich, UK) [19]. Samples were separated by sodium dodecyl sulfate-polyacrylamide gel electrophoresis (SDS-PAGE), electroblotted onto nitrocellulose membrane (GE Healthcare, Little Chalfont, UK), probed with rabbit anti-GFP polyclonal antibody (Santa Cruz Biotechnology, Santa Cruz, USA) followed by HRP-conjugated goat anti-rabbit IgG (Bio-Rad, Hemel Hempstead, UK) and ECL detection (GE Healthcare, Little Chalfont, UK), as described previously [19]. The membrane was stripped and re-probed with mouse 12G10 anti-alpha-tubulin antibody (Developmental Studies Hybridoma Bank, University of Iowa) and HRP-conjugated goat anti-mouse IgG (Bio-Rad) as a loading control [19]. For immunofluorescence studies, transfected cells cultured on glass coverslips were fixed, permeabilized, blocked and incubated with mouse anti-Golgi matrix protein 130 (GM130) antibody (BD Bioscience, Oxford, UK) or mouse anti-PDI antibody (Enzo Life Science, Exeter, UK), followed by AlexaFluor 594 donkey anti-mouse (Invitrogen, Paisley, UK) secondary antibody, as described [17]. Coverslips were mounted onto slides in VECTASHIELD^®^ mounting medium with DAPI (Vector laboratories, Peterborough, UK) and visualized by confocal microscopy using a Leica TCS SP5 confocal system, attached to the DMI 6000 microscope [17].

### Plasma biochemistry

Blood samples were collected from the retro-orbital veins [23] following terminal anaesthesia, and plasma was separated by centrifugation at 3000g for 5 minutes at 4°C. Plasma samples were analysed for total calcium, inorganic phosphate, alkaline phosphatase, albumin, urea, creatinine and glucose, using a Beckman Coulter AU680 semi-automated clinical chemistry analyzer, as described [24]. Plasma calcium was adjusted for variations in albumin concentrations using the formula: ((albumin-mean albumin) x0.02) + calcium) [24].

### Histology and immunohistochemistry

Dissected bones were fixed in 10% formalin and decalcified in formical-4^™^ (Decal Chemical Corporation) for 3 days before embedding in paraffin wax [17]. Bone sections (3–4μm) were stained with Alcian blue 8GX and van Gieson [17]. For immunohistochemical analysis, dewaxed sections were treated with proteinase K for antigen retrieval, before staining with rabbit polyclonal phospho-p38 MAPK (cat # 4631) and phospho-MKK3/MKK6 (cat # 9231) antibodies (Cell Signalling Technology, Danvers, USA) using the ABC staining system (Santa Cruz Biotechnology, Santa Cruz, USA). Digital images were obtained using the Nanozoomer 2.0 Digital Pathology system (Hamamatsu Photonics, Welwyn Garden City, UK).

### Statistical analysis

Mean values and standard deviations (SD) or standard errors of mean (SEM) were calculated and analysis performed using unpaired Student’s t-test for independent samples [19]. The Bonferroni correction for multiple testing was applied [19].

## Results

### Identification of kyphosis (KYLB) mice

**Fig 1:**
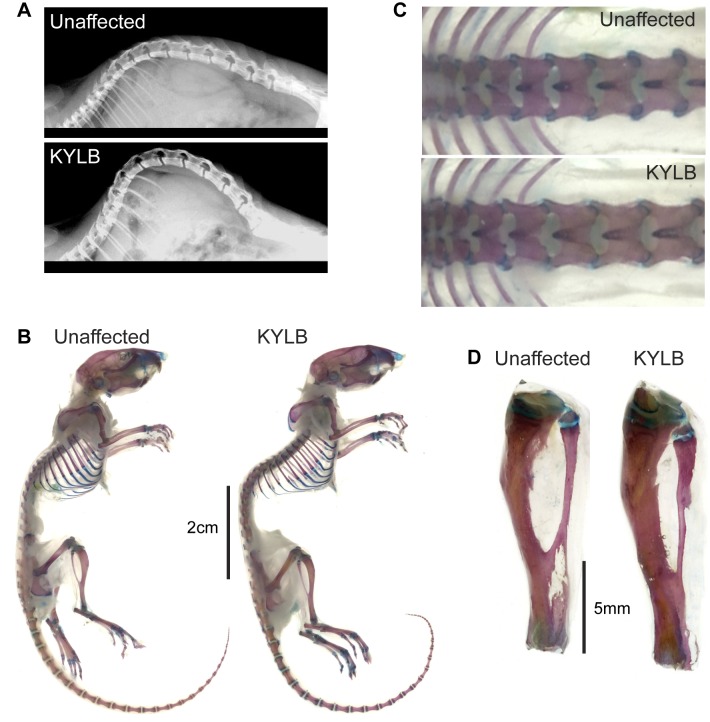
Phenotypic features of KYLB mice. (A) Radiography of adult (8 week old) male KYLB and unaffected male littermate. The KYLB mouse has kyphosis of the thoraco-lumbar spine. (B-D) Alcian blue (cartilage) and Alizarin red (bone) staining of young (4 week old) male KYLB and male unaffected mice. KYLB mice had longer bodies (B), vertebrae (C), and lengthened long bones (tibia shown) (D), when compared to unaffected littermates.

A dysmorphology screen of 10 day old mice revealed that a number of G3 progeny from an ENU-mutagenized BALB/c male mouse had developed curvature of the vertebral column, and these were designated KYLB. Radiography of 8 week old KYLB mice revealed that the vertebral column deformity was due to a thoraco-lumbar kyphosis (Fig 1A) and the severity of the kyphosis was similar in male and female KYLB mice. KYLB mice were observed to have a normal gait without any overt neurological or muscular abnormalities. Alcian blue and Alizarin red S staining to visualize cartilage and bone, respectively, in the skeletons of 4 week old mice confirmed the presence of the thoraco-lumbar kyphosis (Fig 1B). In addition, the KYLB mice were observed to have longer bodies (Fig 1B), an increased length of the vertebrae (Fig 1C) and lengthened long bones (Fig 1D), when compared to their unaffected littermates. The sizes of the calvarial bones were not overtly different in KYLB mice. Breeding of KYLB mice with normal BALB/c mice, and intercrossing the offspring yielded ~23% affected KYLB mice (8 affected (4 males, 4 females) and 27 unaffected (10 males, 17 females)), which is consistent with the expected ~25% of mutants for an autosomal recessive disorder. Thus, KYLB mice represent a model for autosomal recessive thoraco-lumbar kyphosis.

### Mapping of the *Kylb* locus to chromosome 15A1 and identification of an *Npr3* missense mutation

**Fig 2:**
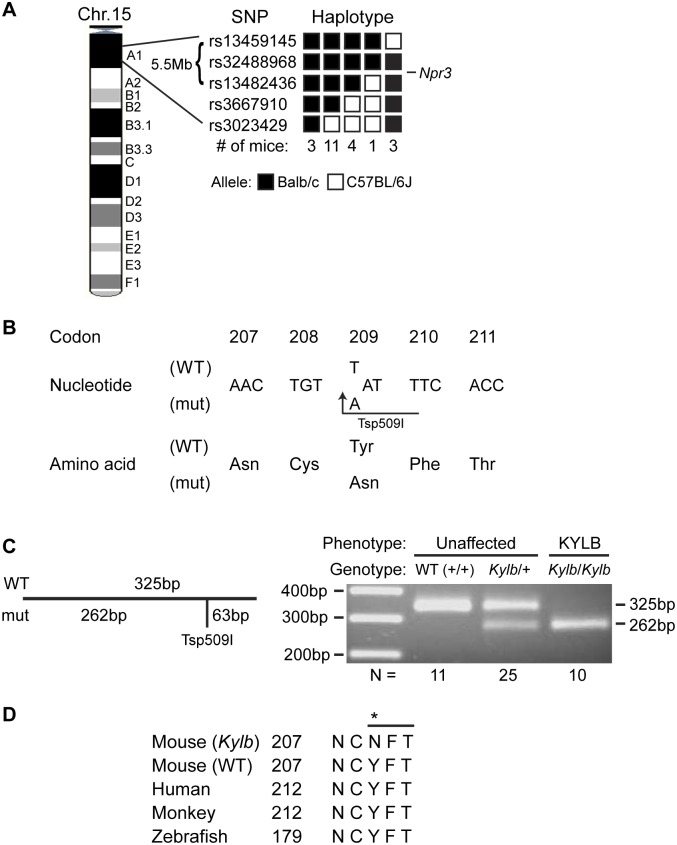
Mapping of *Kylb* locus and identification of *Npr3* mutation. (A) The *Kylb* locus, which originated in a BALB/c ENU-mutagenised male mouse and was hence inherited with the BALB/c alleles, was mapped to a 5.5Mb region flanked by the SNPs rs13459145 and rs13482436 on chromosome 15A1 containing 51 genes, including the most likely candidate, *Npr3*. (B) DNA sequence analysis of the *Npr3* gene revealed a T to A transversion at codon 209 in exon 1, altering the wild-type (WT) sequence TAT, encoding tyrosine (Tyr), to the mutant (m) sequence AAT, encoding asparagine (Asn). The mutation introduced a Tsp509I restriction endonuclease site (/AATT) and this was used to confirm the mutation. (C) PCR primers specific for exon 1 were designed to amplify a 325bp product. Tsp509I did not cleave the WT PCR product but did cleave the mutant PCR product to yield 262bp and 63bp products, as shown by agarose gel electrophoresis of the digested Tsp509I PCR products. KYLB mice were homozygous for the mutant 262bp and 63bp (not shown) products (*Kylb*/*Kylb*), whilst unaffected mice were either homozygous for the 325bp product (WT, +/+) or heterozygous for the wild-type and mutant products (*Kylb*/+). (D) Protein sequence alignment (CLUSTALW) of NPR3 from 4 species revealed that the Tyr (Y) residue is evolutionarily conserved in the NPR3 homologs in mouse, human, monkey and zebrafish. Furthermore, the Asn209 (N) mutant (asterisk) generates a consensus N-linked glycosylation site (N-F-T) (indicated by horizontal line).

Genome-wide analysis using DNA samples from 22 KYLB mice and 91 SNPs mapped the *Kylb* locus to a 5.5Mb region on chromosome 15A1 flanked centromerically by rs13459145 and telomerically by rs13482436 (Fig 2A). This interval contains 51 genes, including the *Npr3* gene, mutations of which have been previously reported to be associated with kyphosis in mice [21, 22, 25]. DNA sequence analysis of the *Npr3* gene in affected mice was therefore undertaken, and this identified a homozygous T to A transversion at codon 209, which altered a tyrosine (Tyr) residue to an asparagine (Asn) residue (Fig 2B). The T to A transversion also generated a Tsp509I restriction endonuclease recognition site that was used to confirm the missense mutation by restriction endonuclease analysis (Fig 2C). Thus, all the affected KYLB mice were homozygous for the mutation (*Kylb*/*Kylb*), whilst unaffected mice were either wild-type (+/+) or heterozygous (*Kylb*/+) (Fig 2C). The mutated nucleotide has a PhyloP score of 2.69, indicating conservation of the nucleotide, and an amino acid sequence alignment of eukaryotic NPR3 homologs revealed that the Tyr209Asn NPR3 mutation alters an evolutionarily conserved tyrosine residue (Fig 2D). Furthermore, the Tyr209Asn mutation introduces a consensus N-linked glycosylation site (N-X-T) of ^209^Asn-Phe-Thr^211^ into the mutant NPR3 protein (Fig 2D).

### Functional characterization of mutant Npr3

**Fig 3:**
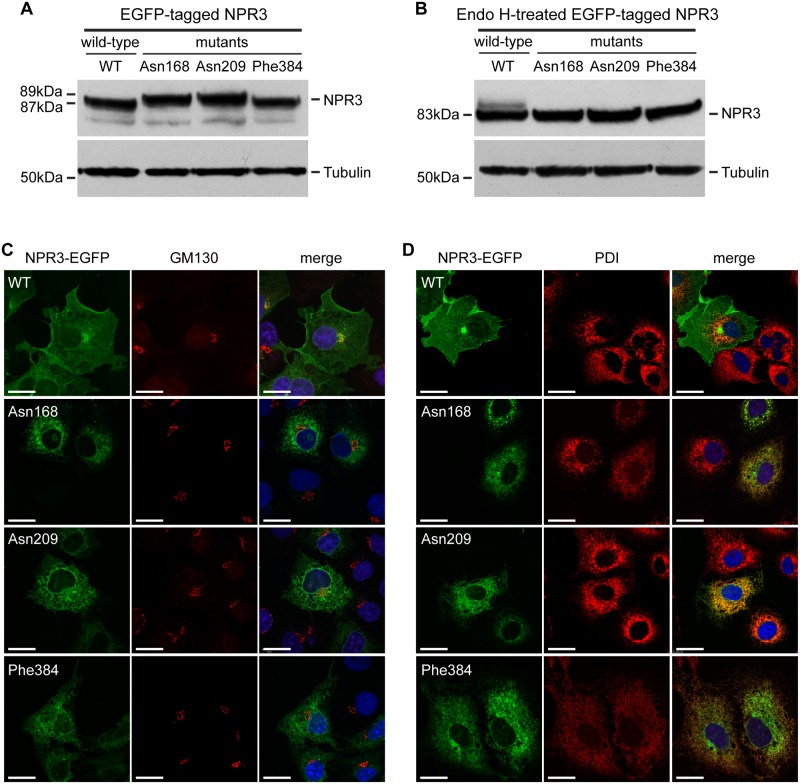
Expression and localization of wild-type and His168Asn, Tyr209Asn and Ile384Phe NPR3 proteins in COS-7 cells. (A) Western blot analysis of cell lysates from COS-7 cells transfected with either the wild-type (WT) or mutant (Asn168, Asn209, or Phe384) EGFP-tagged constructs. The WT and mutant Phe384 NPR3 proteins had molecular weights of 87kDa, whilst the Asn168 and Asn209 NPR3 mutants had a higher molecular weight of 89kDa, consistent with glycosylation at the mutant Asn168 and Asn209 residues. (B) Treatment with Endo H revealed some undigested WT NPR3 protein products whereas all the mutant NPR3 proteins were Endo H-sensitive, suggesting retention in the endoplasmic reticulum (ER). Tubulin was used as a loading control. (C-D) COS-7 cells transiently expressing EGFP-tagged WT or mutant (Asn168, Asn209 and Phe384) NPR3 proteins (green) were counterstained with anti-GM130 antibody (red) which is specific for the Golgi apparatus (C), or anti-PDI antibody (red) which is specific for the ER (D), and DAPI (blue) which is specific for nuclei. WT NPR3 localised to the plasma membrane with some expression in the Golgi apparatus, whereas all the mutant NPR3 proteins co-localised with the ER marker (orange). Scale bars = 20μm.

NPR3 is a type 1 transmembrane protein consisting of a large extracellular domain (476 amino acids), a transmembrane domain (23 amino acids) and a short cytoplasmic domain (37 amino acids) [26], and acts as a decoy receptor for C-type natriuretic peptide (CNP) [27]. The extracellular domain of NPR3 is predicted (NetNGlyc 1.0 Server) to have two consensus sites for N-linked glycosylation (N-X-S/T) at amino acids 81–83 and 389–391. To investigate the functional consequences of the Tyr209Asn NPR3 mutation and to assess the novel putative N-linked glycosylation site, EGFP-tagged wild-type (Tyr209) and mutant (Asn209) *Npr3* cDNA constructs were transfected into COS-7 cells and their expression and subcellular localization assessed. In addition, the *in vitro* consequences of two other ENU-induced NPR3 missense mutations, His168Asn [21] and Ile384Phe [22] that were reported to be associated with kyphosis in Strigosus (Stri) and Eel mice, respectively, but whose cellular effects were not reported, were similarly assessed (Fig 3). Interestingly, the Asn168 NPR3 mutant is also predicted to generate a novel N-linked glycosylation site (^168^Asn-Leu-Thr^170^ (^168^N-L-T^170^)). Western blot analysis of cell lysates obtained from COS-7 cells transiently transfected with wild-type or mutant EGFP-tagged constructs revealed that the Asn168 and Asn209 NPR3 mutants were associated with the presence of a higher molecular weight product of ~89kDa compared to the wild-type and mutant Phe384 NPR3 proteins at ~87kDa (Fig 3A), suggesting that the mutant Asn168 and Asn209 amino acid residues were glycosylated. Treatment of transfected cell lysates with the deglycosylating enzyme, Endo H, which cleaves high mannose N-linked glycans that are introduced in the endoplasmic reticulum (ER), revealed the presence of Endo H-sensitive products in all the cell lysates (Fig 3B), thereby indicating that the wild-type NPR3 and the three mutant (Asn168, Asn209 and Phe384) NPR3 proteins all had high mannose structures, and that all of them had entered the ER lumen. However, lysates from cells expressing wild-type NPR3 protein also had Endo H-resistant products, which were not present in lysates from cells expressing the mutant (Asn168, Asn209 and Phe384) NPR3 proteins (Fig 3B). This suggested that the mutant Asn168, Asn209 and Phe384 NPR3 proteins did not undergo the complex oligosaccharide modifications that occur in the Golgi apparatus, but may instead be retained in the ER. Immunofluorescence and confocal microscopy studies of the transiently transfected COS-7 cells confirmed this prediction. Thus, wild-type NPR3 protein was predominantly located at the plasma membrane, with some wild-type NPR3 protein also located in the Golgi apparatus, which was indicated by co-localization with the Golgi-matrix protein, GM130 (Fig 3C). By contrast, the mutant Asn168, Asn209 and Phe384 NPR3 proteins were mislocalized and retained in the ER (Fig 3D), as indicated by co-localization with protein disulphide isomerase (PDI) (Fig 3D).

### Phenotyping and clinical chemistry analysis of 12 week old wild-type, *Kylb*/+ and *Kylb*/*Kylb* mice

*Kylb*/+ mice were intercrossed to generate wild-type, *Kylb*/+ and *Kylb*/*Kylb* mice. The crown to rump length of *Kylb*/*Kylb* male and female adult mice, aged 12 weeks, was ~12% greater than that of the *Kylb*/+ and wild-type littermates (p<0.01), although the body weights were similar in *Kylb*/*Kylb* and wild-type littermates (Table 1). Femoral lengths in *Kylb*/*Kylb* mice were greater than 2 standard deviations longer than unaffected (wild-type and *Kylb*/+) littermates (S1 Fig). Whole body DXA analysis revealed that *Kylb*/*Kylb* mice, when compared to *Kylb*/+ and wild-type littermates, had significant increases in bone area by ~22% (p<0.001) in females and ~12% (p<0.05) in males, increased bone mineral content by ~18% (p<0.05) in females, and reduced percentage body fat by ~28% (p<0.05) in females (Table 1). Plasma biochemical analysis of 12 week old wild-type (males n = 8 and females n = 8), *Kylb*/+ (males n = 13 and females n = 13) and *Kylb*/*Kylb* (males n = 7 and females n = 4) mice did not reveal any alterations in the levels of urea, creatinine, calcium, phosphate or albumin (S1 Table). However, female, but not male *Kylb*/*Kylb* mice had significantly elevated alkaline phosphatase (ALP) levels (mean ± SD = 127 ± 21 U/l, p<0.05) compared to *Kylb*/+ (98 ± 12 U/l) and wild-type littermates (93 ± 15 U/l), suggesting increased bone turnover.

**Table 1:** Body weight, length and DXA analysis of 12 week old mice.

	Females			Males		
	Wild-type (n = 6)	*Kylb*/*+* (n = 11)	*Kylb*/*Kylb* (n = 7)	Wild-type (n = 7)	*Kylb*/*+* (n = 11)	*Kylb*/*Kylb* (n = 4)
Body weight (g)	23.7 ± 1.7	24.5 ± 2.9	23.7 ± 1.4	30.5 ± 1.5	32.3 ± 2.5	31.5 ± 2.5
Body length (cm)^a^	9.1 ± 0.4	9.3 ± 0.2	10.2 ± 0.2^$^	9.5 ± 0.2	9.9 ± 0.4	10.6 ± 0.5**
Bone area (cm^2^)	8.3 ± 0.6	8.7 ± 0.8	10.1 ± 0.4^$^	9.0 ± 0.4	9.4 ± 0.6	10.1 ± 0.7*
BMC (mg)	413 ± 44	430 ± 51	488 ± 22*	493 ± 38	517 ± 44	513 ± 60
BMD (mg/cm^2^)	49.6 ± 2.1	49.2 ± 2.0	48.2 ± 1.9	54.9 ± 2.9	55.1 ± 1.9	50.9 ± 3.0
Lean mass (g)	18.3 ± 1.0	18.6 ± 1.7	18.8 ± 1.1	24.5 ±1.4	25.0 ± 1.8	25.3±1.9
Fat mass (g)	4.5 ± 1.1	5.1 ± 1.6	3.1 ± 0.9	3.8 ± 1.0	5.1 ± 1.7	4.9 ± 0.4
Fat content (%)	19.4 ± 3.5	21.3 ± 5.2	13.9 ± 2.8*	13.4 ± 2.4	16.6 ± 4.1	16.3 ± 1.2

Values are expressed as mean ± SD.

^a^ Body length measured from crown to rump. BMC, bone mineral content; BMD, bone mineral density.

*p<0.05,

**p<0.01,

^$^p<0.001.

### KYLB mice have delayed endochondral ossification and increased activation of p38 mitogen-activated protein kinase (MAPK) signaling

**Fig 4:**
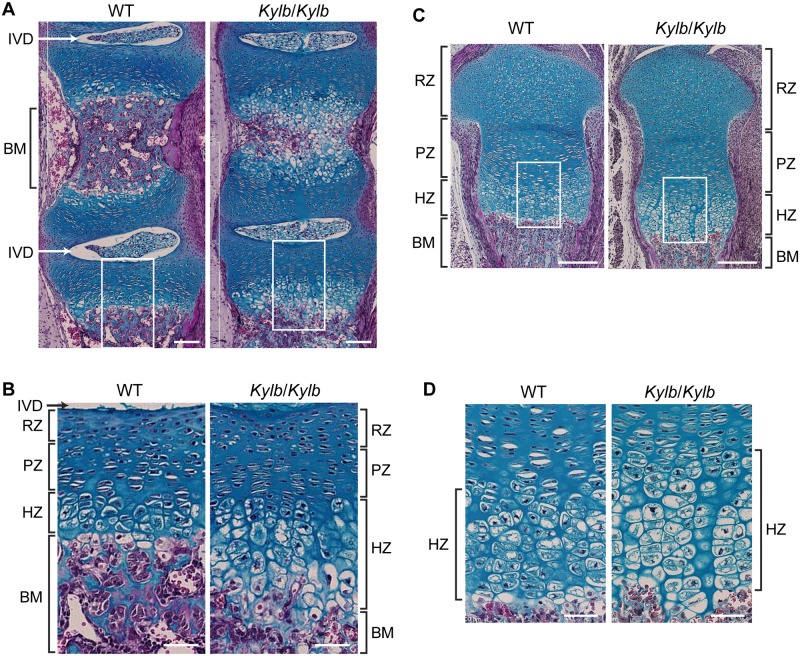
Histomorphometric analysis of vertebral and tibial growth plates. Alcian blue and van Gieson staining of lumbar vertebrae (A, B) and tibiae (C, D) from 1 day old male mice showing reduced bone matrix (BM) formation (red stain) between intervertebral discs (IVD) and distal tibia in *Kylb*/*Kylb* mice compared to wild-type (WT) littermates. White boxes delineate detailed views shown in (B) and (D) of growth plates, showing expansion of the hypertrophic zone (HZ) in *Kylb*/*Kylb* mice compared to WT littermates. The resting (RZ) and proliferative (PZ) zones in the *Kylb*/*Kylb* and WT mice appeared to be similar and not affected. Scale bars = 100μm (A); 50μm (B, D); 200μm (C).

**Fig 5:**
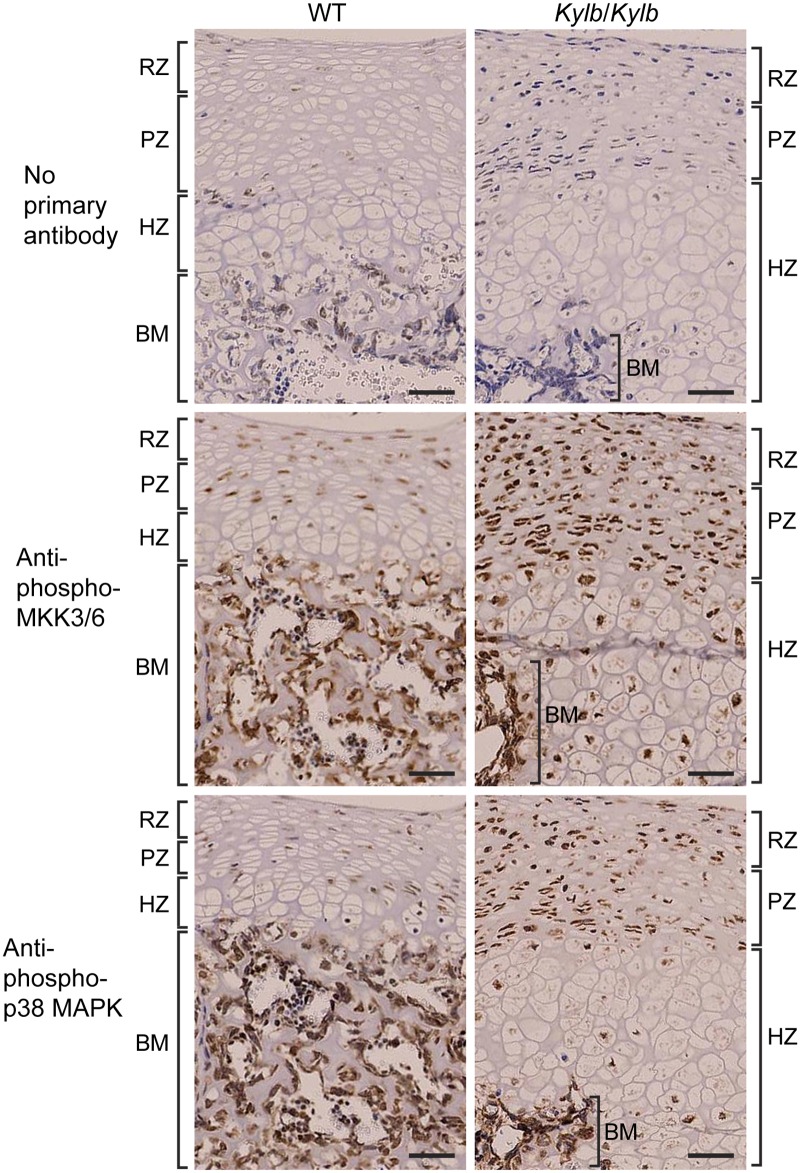
p38 MAPK signaling in vertebral growth plates. Vertebral sections from 1 day old wild-type (WT) and *Kylb/Kylb* male mice were stained with antibodies against phosphorylated p38 MAPK and MKK3/MKK6. Vertebrae from *Kylb*/*Kylb* mice had increased staining in the resting (RZ), proliferative (PZ) and hypertrophic (HZ) zones, but not in the bone matrix (BM), when compared to vertebrae from WT littermates. Scale bars = 50μm.

To investigate the basis of the greater lengths of the vertebrae (Fig 1C) and long bones (Fig 1D) in *Kylb*/*Kylb* mice, lumbar vertebral (Fig 4A and 4B) and tibial (Fig 4C and 4D) sections from 1 day old mice were stained with Alcian blue which stains cartilage blue, and van Gieson which stains osteoid in the bone matrix red. Bone matrix formation was observed to be prominent in the central region of lumbar vertebrae in wild-type mice, but was deficient in *Kylb*/*Kylb* mice, in whom the central region mainly consisted of cartilage (Fig 4A). The hypertrophic zone (HZ) in *Kylb*/*Kylb* mice was expanded ~2-fold in the vertebrae (Fig 4B). Similarly, in the tibiae of *Kylb*/*Kylb* mice the HZ was expanded when compared to those from wild-type mice (Fig 4C and 4D). Thus, there is likely a delay in endochondral ossification in the vertebrae and tibiae of *Kylb*/*Kylb* mice. NPR3 is a decoy receptor for C-type natriuretic peptide (CNP), and functional loss of NPR3 may result in increased CNP signaling [25]. CNP-stimulated expansion of the hypertrophic zone of the growth plate in a tibia organ culture system has been reported to be dependent on the activation of the p38 mitogen-activated protein kinase (MAPK) signaling pathway [27], and we therefore explored the role of the p38 MAPK signaling pathway in causing the expansion of the vertebral growth plate. Immunohistochemical analysis of lumbar vertebral sections from 1 day old mice to detect phosphorylated (active) p38 MAPK revealed that *Kylb*/*Kylb* mice, when compared to wild-type littermates, had an increase in phosphorylated p38 MAPK in the resting, proliferative and hypertrophic zones, but not bone matrix, of their vertebrae (Fig 5). Furthermore, phosphorylation of the upstream activators of p38 MAPK, MAPK kinase 3/ MAPK kinase 6 (MKK3/MKK6) was also increased in the resting, proliferative, and hypertrophic zones in vertebrae of *Kylb*/*Kylb* mice compared to wild-type littermates (Fig 5). Thus, there is an increased activation of the p38 MAPK signaling pathway in *Kylb/Kylb* vertebrae.

## Discussion

We have established an ENU-induced mutant mouse model, KYLB, for autosomal recessive kyphosis due to a novel Tyr209Asn mutation in NPR3. In addition, our studies have revealed that this Tyr209Asn NPR3 mutation generates a new N-linked glycosylation site, which is utilised and results in the ER retention of the mutant NPR3 protein. Finally, our investigation shows that the kyphosis, due to lengthening of vertebrae, is associated with a delay of endochondral ossification and activation of the p38 MAPK signaling pathway. Interestingly, this did not prevent KYLB mice from attaining a normal BMD by the age of 12 weeks, indicating that the process of endochondral ossification was not defective in KYLB mice but only delayed.

The phenotypic features of the KYLB mouse have both similarities and differences compared to the other 5 mouse models due to NPR3 abnormalities that have been previously described (Table 2). These mouse models are: a *Npr3* null mouse generated by homologous recombination [25]; two spontaneously occurring models referred to as Longjohn (Lgj) and Longjohn-2J (Lgj^2J^) [21], which are due to a 36bp in-frame deletion and a nonsense Gln95Stop NPR3 mutation, respectively; and two ENU-induced mutations, referred to as Strigosus (Stri) [21] and Eel [22] which are due to His168Asn and Ile384Phe NPR3 missense mutations, respectively. Similarities between KYLB mice and previously reported models include the occurrence of kyphosis and lengthening of the body and long bones. However, there are also important differences, which include: the occurrence of arachnodactyly in the Lgj, Lgj^2J^ and Stri models [21]; sacral/tail kinks in the Lgj, Lgj^2J^, Stri and *Npr3* null mice [21, 25]; and premature death in Lgj^2J^ and *Npr3* null mice [21, 25]. In addition, vertebral enlargement, which was present in KYLB mice (Fig 1C), was reported in Stri mice, but not in the other models; and delayed endochondral ossification, which was found in KYLB mice (Fig 4), was reported in Stri and *Npr3* null mice, but not in the other models (Table 2). The basis of these differences may in part be due to the intracellular effects of the NPR3 mutant proteins, and this is well illustrated by our *in vitro* expression studies of the three NPR3 missense mutants, which revealed that the Asn168 and Asn209 mutations resulted in abnormal N-linked glycosylation with ER retention, whereas the Phe384 mutation was not associated with abnormal N-glycosylation but was retained in the ER. Thus, these three missense NPR3 mutant proteins would result in defective trafficking and an absence of functional NPR3 at the plasma membrane, a situation that is also likely to be the case in the *Npr3* null mice. This may help to explain the close similarities in the phenotypes of these mutant mice (Table 2). In contrast, it has been reported that the 12 amino acid in-frame deletion giving rise to Lgj may result in defective ligand binding capability of the mutant proteins at the plasma membrane [21], and this may in part explain the phenotypic differences between the Lgj mouse and the KYLB, Stri and *Npr3* null mice. During the course of our studies, another model of kyphosis designated Skm1, due to an Ile384Asn NPR3 mutation was reported [28] (Table 2); however the Asn384 mutation does not introduce a consensus N-linked glycosylation site, similarly to the Eel NPR3 mutation, and thus is likely to be retained in the ER but without abnormal N-linked glycosylation. Further characterisation of the bone alterations in KYLB mice by DXA analysis revealed an increase in bone area, which was associated with a corresponding increase in bone mineral content only in females. These changes were not reported in any of the Stri, Lgj, Lgj^2J^, *Npr3* null, Eel or Skm1 mouse models. The reason for the gender-specific differences in some of the bone parameters in female but not male KYLB mice remains to be elucidated; however, other mouse models for skeletal disorders have also demonstrated gender-specific effects [17].

**Table 2:** Comparison of mouse models with kyphosis due to *Npr3* mutations.

Mouse model	Stri	Lgj	Lgj^2J^	Npr3 null	Eel	Skm1	KYLB
Derivation	ENU	SPO	SPO	HR	ENU	ENU	ENU
Mutation	His168Asn	36bp del	Gln95Ter	Del exon1	Ile384Phe	Ile384Asn	Tyr209Asn
**Phenotypic features**							
Kyphosis	+	+	+	+	+	+	+
Elongated body	+	+	+	+	+	+	+
Elongated long bones	+	+	+	+	+	+	+
Enlarged vertebrae	+	?	?	?	?	?	+
Blood pressure	?	?	?	reduced	?	+	?
Urine concentration	?	?	?	reduced	?	+	?
Delayed endochondral ossification	+	?	?	+	?	-	+
Arachnodactyly	+	+	+	?	?	?	–
Sacral/tail kinks	+	+	+	+	?	?	–
Premature death	?	–	+	+	?	?	–
Fate of mutant NPR3	ER retained*	?	?	absent	ER retained*	?	ER retained*
Reference	[21]	[21]	[21]	[25]	[22]	[28]	This study

SPO, spontaneous; HR, homologous recombination; Del, deletion;?, unknown; +, present;–, not present;

*, reported in this study

**Fig 6:**
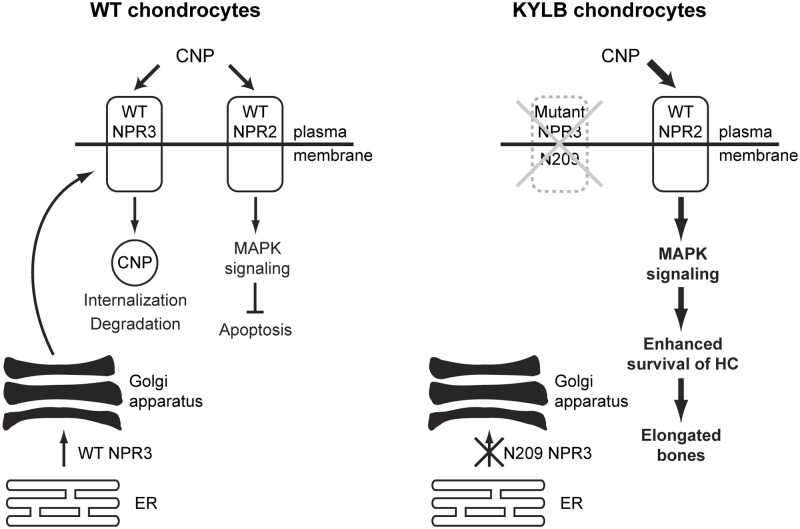
Schematic model illustrating the role of NPR3 as a decoy receptor. In wild-type (WT) chondrocytes, NPR3 binds CNP thereby promoting its internalization and degradation, and reducing its availability to bind to NPR2 to stimulate the MAPK signaling pathway, whose activation inhibits termination of hypertrophic chondrocytes (HC) and bone formation. In KYLB mice who have a homozygous Tyr209Asn NPR3 mutation, NPR3 is abnormally glycosylated and retained in the ER. The absence of NPR3, and its loss as a decoy receptor for CNP, results in a greater availability of CNP to bind to NPR2, leading to increased MAPK signaling and enhanced survival of hypertrophic chondrocytes, resulting in the lengthening of bones.

The absence of functional NPR3 at the plasma membrane may help to explain the observed delay in endochondral ossification that likely involves increased activation of the p38 MAPK signaling pathway. Thus, NPR3, which has a wide tissue expression pattern including chondrocytes [29] and likely functions as a clearance receptor for natriuretic peptides [30], has emerged as a key regulator of endochondral bone growth through its action on locally produced CNP [27, 29]. Binding by CNP to its receptor, NPR2 [31], stimulates endochondral ossification and bone growth likely by actions on hypertrophic chondrocytes, which include an increase in their production and a delay in their replacement with bone [27]. Indeed, *in vitro* studies have demonstrated that CNP can stimulate longitudinal growth of cultured mouse tibiae [27], and that these effects are mediated by the p38 MAPK signaling pathway [27, 32]. Furthermore, *in vivo* studies have shown that: dwarfism occurs in CNP-deficient mice [29], and in mice and patients with *Npr2* mutations [33, 34]; mice overexpressing CNP have skeletal overgrowth [35]; targeted overexpression of CNP in chondrocytes can rescue dwarfism in a mouse model of achondroplasia, through a MAPK-dependent pathway [36]; and genome-wide association studies in humans have identified significant associations between the CNP signaling pathway and measures of height and skeletal size [37, 38]. Thus, these *in vitro* and *in vivo* findings demonstrate an important role for CNP and NPR2 in activating MAPK signaling and in delaying hypertrophic chondrocyte replacement by bone matrix [27] (Figs 4 and 5). Within this scheme, the role of NPR3 is to act as a decoy receptor that binds CNP and promotes its internalization and degradation, thereby reducing the bioavailability of CNP and hence its stimulatory effect on endochondral bone growth [39]. Thus, mutations of NPR3, which prevent its appearance at the plasma membrane (Fig 3C), will result in decreased clearance of CNP, leading to increased activation of p38 MAPK signaling, as demonstrated by our observations of an increased expression of phosphorylated MKK3/MKK6 and p38 MAPK in the vertebral growth plates from KYLB mice (Fig 5). We therefore propose a molecular and cellular model (Fig 6) to explain the kyphosis that results from the NPR3 mutation in the KYLB mice, as follows. In the hypertrophic chondrocytes of wild-type mice, NPR3 is synthesised in the ER, post-translationally modified with high mannose N-linked glycans, and transported to the Golgi whereby it undergoes further processing with addition of complex oligosaccharides residues. The mature NPR3 is then transported to the plasma membrane where it both terminates p38 MAPK signalling in the hypertrophic chondrocytes, and regulates the availability of CNP to signal through the MKK3/MKK6 and p38 MAPK signaling pathway in resting and proliferating chondrocytes. However, in the hypertrophic chondrocytes of KYLB mice, mutant NPR3 is synthesised in the ER and post-translationally modified with high mannose N-linked glycans, but is not transported to the Golgi apparatus or the plasma membrane. Thus, uncleared CNP is available to bind to NPR2 and persistently stimulate p38 MAPK signaling in resting, proliferative and hypertrophic chondrocytes, thereby delaying bone matrix formation from hypertrophic chondrocytes, whose enhanced survival results in a longer period of growth and elongated bones. With the exception of mice overexpressing CNP [35], the other reported mouse models for kyphosis do not appear to have the same pathogenesis as mice with mutations in Npr3, further emphasizing the multiple etiologies of kyphosis.

In conclusion, we have established a novel mouse model for kyphosis associated with lengthened vertebrae and long bones that harbors a Tyr209Asn mutation in NPR3. Vertebrae in KYLB mice demonstrated delayed ossification, likely due to increased activation of the p38 MAPK pathway that is activated by CNP. This is consistent with the role of NPR3 as a decoy receptor for CNP and our studies have further elucidated the role of CNP and NPR3 in endochondral ossification.

## Supporting Information

S1 Fig Femoral lengths in 12 week old mice. Femoral lengths of 4 week old wild-type (WT), *Kylb*/+ and *Kylb/Kylb* male and female mice. Femora from *Kylb*/*Kylb* mice were more than 2 standard deviations (SD) longer than those from unaffected (WT and *Kylb*/+) mice. Individual squares (males) and circles (females) represent individual mice; the bars represent the mean ± SD of unaffected mice. Open symbols represent WT mice, gray symbols represent *Kylb*/+ mice, and black symbols represent *Kylb*/*Kylb* mice.
(TIF)

S1 Table Plasma biochemical analysis of 12 week old wild-type, *Kylb*/+ and *Kylb*/*Kylb* mice. Values are expressed as mean ± SD. Corr. Ca: corrected calcium; Pi: inorganic phosphate.
(DOCX)
