# Supplementary figures and images for: Mice with an N-Ethyl-N-Nitrosourea (ENU) Induced Tyr209Asn Mutation in Natriuretic Peptide Receptor 3 (NPR3) Provide a Model for Kyphosis Associated with Activation of the MAPK Signaling Pathway
Source: PLoS One. 2016 Dec 13;11(12):e0167916. doi: 10.1371/journal.pone.0167916 (PMC5154531; doi:10.1371/journal.pone.0167916)

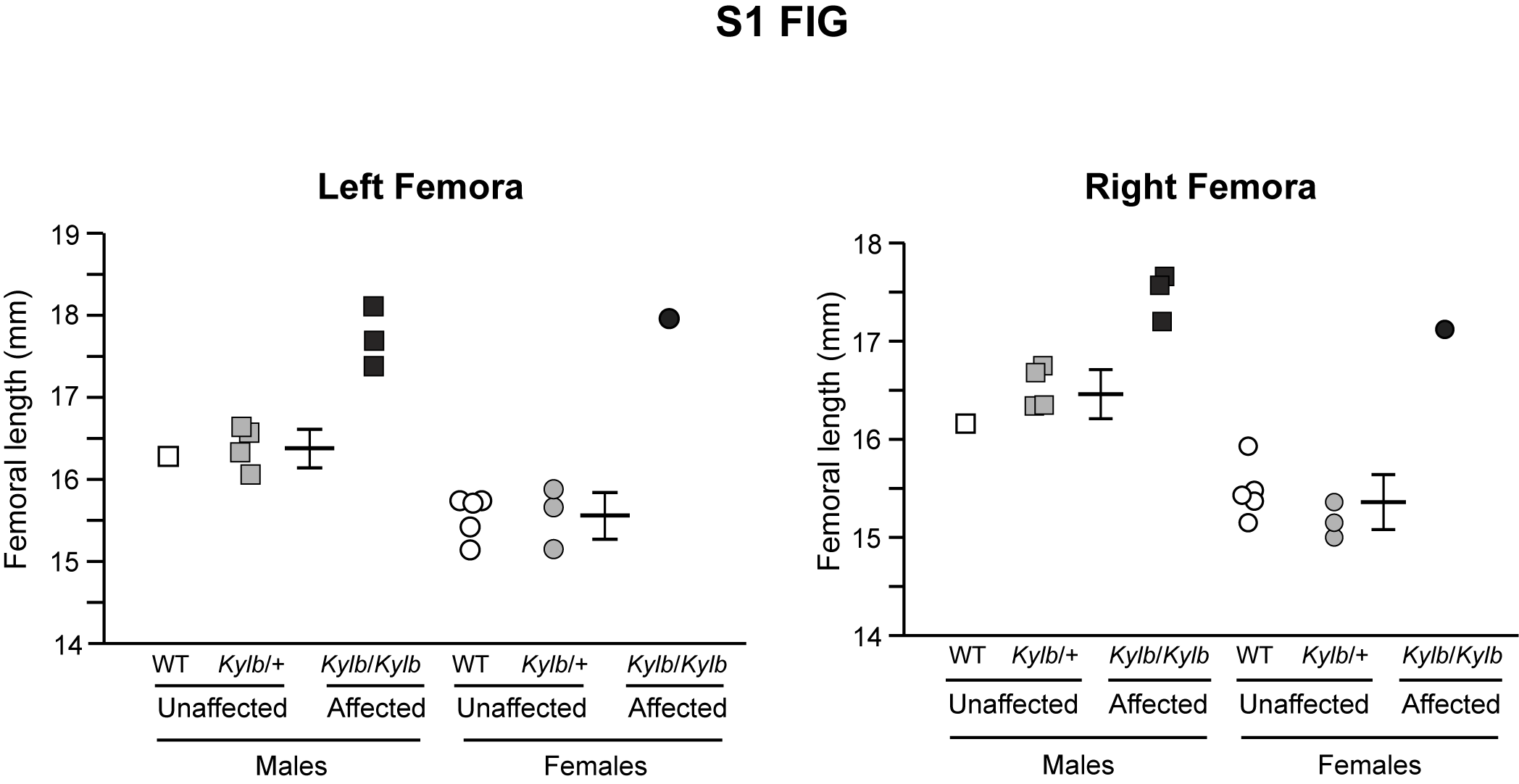

Supplement: S1 Fig — Femoral lengths of 4 week old wild-type (WT), Kylb/+ and Kylb/Kylb male and female mice. Femora from Kylb/Kylb mice were more than 2 standard deviations (SD) longer than those from unaffected (WT and Kylb/+) mice. Individual squares (males) and circles (females) represent individual mice; the bars represent the mean ± SD of unaffected mice. Open symbols represent WT mice, gray symbols represent Kylb/+ mice, and black symbols represent Kylb/Kylb mice. (TIF) [file pone.0167916.s001.tif]
