# Supplementary material for: Mice with an N-Ethyl-N-Nitrosourea (ENU) Induced Tyr209Asn Mutation in Natriuretic Peptide Receptor 3 (NPR3) Provide a Model for Kyphosis Associated with Activation of the MAPK Signaling Pathway
Source: PLoS One. 2016 Dec 13;11(12):e0167916. doi: 10.1371/journal.pone.0167916 (PMC5154531; doi:10.1371/journal.pone.0167916)
Supplement: S1 Table — Values are expressed as mean ± SD. Corr. Ca: corrected calcium; Pi: inorganic phosphate. (DOCX) [file pone.0167916.s002.docx]

**S1 Table. Plasma biochemical analysis of 12 week old wild-type, *Kylb*/*+* and *Kylb*/*Kylb* mice**

|  | **Females** | | | **Males** | | |
| --- | --- | --- | --- | --- | --- | --- |
|  | Wild-type (n=8) | *Kylb*/*+* (n=13) | *Kylb*/*Kylb* (n=7) | Wild-type (n=8) | *Kylb*/*+* (n=13) | *Kylb*/*Kylb* (n=4) |
| Urea (mmol/l) | 8.8 ± 2.4 | 10.3 ± 1.5 | 9.8 ± 1.5 | 9.6 ± 1.5 | 10.0 ± 2.2 | 10.4 ± 2.3 |
| Creatinine (µmol/l) | 13.3 ± 2.1 | 14.6 ± 1.6 | 12.2 ± 2.2 | 10.8 ± 1.1 | 11.7 ± 2.1 | 11.2 ± 2.8 |
| Corr. Ca (mmol/l) | 2.3 ± 0.2 | 2.2 ± 0.2 | 2.1 ± 0.3 | 2.2 ± 0.2 | 2.0 ± 0.4 | 2.2 ± 0.3 |
| Pi (mmol/l) | 2.6 ± 0.6 | 2.4 ± 0.5 | 2.7 ± 0.9 | 2.3 ± 0.4 | 2.1 ± 0.4 | 2.8 ± 0.5 |
| Albumin (g/l) | 26.5 ± 1.9 | 26.5 ± 1.6 | 25.8 ± 1.3 | 23.0 ± 1.5 | 22.5 ± 1.8 | 23.0 ± 2.4 |

Values are expressed as mean ± SD. Corr. Ca: corrected calcium; Pi: inorganic phosphate.
